# DeepPhosPPI: a deep learning framework with attention-CNN and transformer for predicting phosphorylation effects on protein–protein interactions

**DOI:** 10.1093/bib/bbaf462

**Published:** 2025-09-07

**Authors:** Yinyin Gong, Rui Li, Yan Liu, Jilong Wang, Danny Z Chen, Chee Keong Kwoh

**Affiliations:** College of Computer Science and Electronic Engineering, Hunan University, Changsha, 410083, China; College of Computing and Data Science, Nanyang Technological University, 639798, Singapore; College of Computer Science and Electronic Engineering, Hunan University, Changsha, 410083, China; College of Computer Science and Electronic Engineering, Hunan University, Changsha, 410083, China; Research and Innovation Center for Convergence of Automobile and Cyberspace, Research Institute of Hunan University in Chongqing, Chongqing, 401120, China; Peng Cheng Laboratory, Shenzhen, 518066, China; Department of Computer Science and Engineering, University of Notre Dame, Notre Dame, IN 46556, USA; College of Computing and Data Science, Nanyang Technological University, 639798, Singapore

**Keywords:** deep learning, ensemble model, phosphorylation, protein–protein interaction, protein sequence

## Abstract

Protein phosphorylation regulates protein function and cellular signaling pathways, and is strongly associated with diseases, including neurodegenerative disorders and cancer. Phosphorylation plays a critical role in regulating protein activity and cellular signaling by modulating protein–protein interactions (PPIs). It alters binding affinities and interaction networks, thereby influencing biological processes and maintaining cellular homeostasis. Experimental validation of these effects is labor-intensive and expensive, highlighting the need for efficient computational approaches. We propose DeepPhosPPI, the first sequence-based deep learning framework for phosphorylation effects on PPIs prediction, which employs the pre-trained protein language model for feature embedding, with ProtBERT and ESM-2 as alternative backbone encoders. By combining attention-based convolutional neural network and Transformer models, DeepPhosPPI accurately predicts phosphorylation effects. The experimental results show that DeepPhosPPI consistently outperforms state-of-the-art methods in multiple tasks, including functional sites identification and regulatory effect classification.

## Introduction

Protein phosphorylation is a key post-translational modification (PTM) that regulates protein function and cellular signaling by transferring phosphate groups to serine (S), threonine (T), or tyrosine (Y) residues [1, 2]. It plays vital roles in biological processes such as protein–protein interactions (PPIs) and transcriptional regulation [3, 4]. As essential components of signaling networks, PPIs maintain cellular homeostasis and mediate diverse biological activities [5]. Dysregulated phosphorylation can disrupt PPIs, contributing to diseases such as cancer and neurodegenerative disorders [6, 7]. Therefore, investigating how phosphorylation regulates PPIs is crucial for uncovering disease mechanisms and developing targeted therapies.

Traditional experimental methods for identifying phosphorylation effects on PPIs, such as Co-IP, pull-down, SPR, and FRET [8], are costly, time-consuming, and limited for large-scale proteomic studies, making computational prediction approaches critical to tackle this challenge. Existing computational methods are generally classified into structure-based and sequence-based approaches. Structure-based methods rely on high-resolution 3D structures and are commonly employed to quantify binding free energy and interfacial potential, such as HawkDock [9], FoldX [10], and Betts [11]. Although these methods are effective, they exhibit limited predictive power for disordered proteins or proteins lacking structural information and are computationally intensive. In comparison, sequence-based methods exhibit significant advantages. Recent research employed machine learning model LGBM [12], demonstrating promising performance in predicting phosphorylation effects on PPIs. However, existing methods are highly dependent on manually engineered features and focus only on local features, neglecting their ability to capture the biochemical properties and complex global sequence dependencies [13].

In recent years, the rapid development of deep learning (DL) methods has provided new opportunities to predict phosphorylation effects on PPIs [13, 14]. Compared to classical machine learning approaches, DL models autonomously extract rich feature representations and capture complex dependencies within protein sequences [15]. DL methods have been effectively applied to various protein sequence analysis tasks, including protein structure prediction (e.g., AlphaFold) [16]) and protein interaction site prediction (e.g., CLPPIS [17] and EnsemPPIS [18]). However, accurately predicting phosphorylation regulatory effects remains challenging because it requires both identifying functional sites and classifying their effects on PPIs, necessitating deep exploration of complex sequence-embedded features.

**Figure 1:**
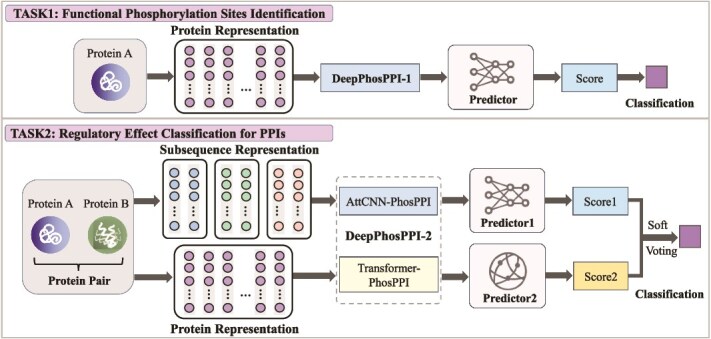
Overview of the DeepPhosPPI framework. Task 1 predicts functional phosphorylation sites using DeepPhosPPI-1, a CNN-based model with attention mechanism. Task 2 classifies regulatory effects on PPIs using DeepPhosPPI-2, which ensembles AttCNN-PhosPPI and Transformer-PhosPPI through soft voting.

We propose DeepPhosPPI, the first **Deep** learning framework for identifying the effects of **Phos**phorylation on **P**rotein–**P**rotein **I**nteractions (illustrated in Fig. 1). DeepPhosPPI is developed for two primary tasks: identification of functional phosphorylation sites and classification of their regulatory effects on PPIs. To efficiently extract features from protein sequences, we leverage pre-trained protein language models (PLMs), with flexible support for ProtBERT [19] or ESM-2 [20], which encodes sequences into high-dimensional embeddings that capture contextual dependencies and biochemical properties. Additionally, to further extract key features associated with phosphorylation and PPIs, we design a novel sliding window strategy, called STY-Sliding Window, which is selectively applied to phosphorylation-specific residues (S, T, Y). DeepPhosPPI first employs an attention-based convolutional neural network (CNN) to focus on the contextual information of the target and their adjacent residues, extracting features related to functional phosphorylation sites. Subsequently, to predict the regulatory effects of phosphorylation sites on PPIs, including enhancement, inhibition, and no impact, our framework integrates the attention-based CNN and a Transformer. CNN extracts critical contextual information by focusing on local features, while Transformer captures global dependencies and long-range relationships. Finally, the predictions of the CNN and Transformer models are fused by using a soft voting ensemble to generate the final results.

## Material and method

### Problem definition

To comprehensively investigate phosphorylation-mediated regulation, we divide the problem into two consecutive tasks of binary classification. The first focuses on predicting functional phosphorylation sites within input protein sequences. The second aims to determine the regulatory effects of phosphorylation at these functional sites on interactions between specific protein pairs. Let $A = \{a_{1}, a_{2}, \ldots , a_{L_{A}}\}$ and $B = \{b_{1}, b_{2}, \ldots , b_{L_{B}}\}$ denote two protein sequences, where $L_{A}$ and $L_{B}$ are the respective sequence lengths. Define $P = \{p_{i} \mid a_{p_{i}} \in \{S, T, Y\}, 1 \leq p_{i} \leq L_{A}\}$ as the set of potential phosphorylation site positions in protein sequence $A$, where $a_{p_{i}}$ represents the residue at position $p_{i}$, belonging to S, T, or Y.

#### TASK1: functional phosphorylation sites identification

The first task identifies functional phosphorylation sites from all $S, T, Y$ residues. It is formalized as a mapping function $F_{1}: p_{i} \to s_{i}$, where $s_{i} \in \{0, 1\}$ represents whether the residue $a_{p_{i}}$ at position $p_{i}$ is functional ($s_{i} = 1$ ) or not ($s_{i} = 0$).

#### TASK2: regulatory effect classification for PPIs

The second task predicts the regulatory effects of functional phosphorylation sites on PPIs. Let $p \in P$ denote a functional site on protein $A$. It is formalized as a mapping function $F_{2}: (A, B, p) \to y$, where $y \in \{0, 1\}$ specifies the regulatory effect. Specifically, $y = 1$ signifies that phosphorylation at $a_{p}$ in protein $A$ enhances the interaction between proteins $A$ and $B$, while $y = 0$ means it inhibits the interaction.

### Data preparation

#### DatasetA

Dataset A is constructed for the identification of functional phosphorylation sites. 1,099 proteins with recorded phosphorylation events are selected from the PTMint database [21]. We extract 2,154 experimentally validated functional sites as positive samples, while 122,410 non-functional S, T, and Y residues are collected as initial negative samples. All positive samples are retained to fully utilize the distinctive information of these phosphorylation sites. Redundancy in negative samples is reduced using PSI-CD-HIT [22] with a sequence similarity threshold of 20%, resulting in 45,397 negative samples. Subsequently, we perform stratified random sampling to select 2,154 negative samples to balance the dataset. Finally, the dataset consists of 4,308 samples and is divided into training and testing sets at an 8:2 ratio, containing 3,444 and 864 samples, respectively.

#### DatasetB

DatasetB is built for regulatory effect classification of functional phosphorylation sites on PPIs. It comprises 3,122 experimentally validated PPI records from the PTMint database, each containing a protein pair, a phosphorylation site, and its regulatory function. Enhancement indicates that phosphorylation increases the binding affinity between proteins, while inhibition indicates a reduction in binding affinity. To better evaluate model performance and compare with existing methods, we use the Betts dataset [11] (comprising 306 records, including 217 enhancement and 89 inhibition cases) as an independent benchmark test set. In addition, we construct an independent test set, Dset_308, comprising 308 experimentally validated records (including 213 enhancement and 95 inhibition cases) from the PTMint database, to further evaluate the generalization ability of our model. Records overlapping with the test datasets are removed, resulting in a final set of 2,775 records for model training, including 1,930 positive (enhancement) and 845 negative (inhibition) samples. Statistics of the datasets are given in Supplementary Material Table S1.

### Feature representation

**Figure 2:**
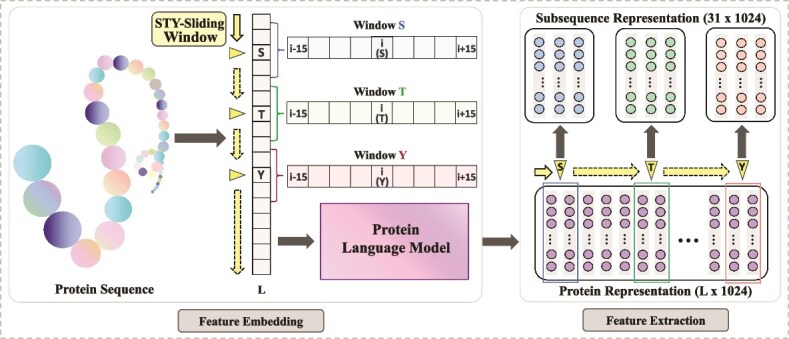
The process of feature representation. It consists of Feature Embedding and Feature Extraction. The protein sequence is embedded using the pre-trained PLM to generate global feature representation. Then, STY-Sliding Window extracts local features from this embedding, focusing on potential phosphorylation residues to capture their contextual information. The embedding dimension is 1024 for ProtBERT (shown) and 1280 for ESM-2.

We employ a hierarchical feature extraction strategy combined with PLM to obtain comprehensive feature representations from protein sequences, as illustrated in Fig. 2.

#### Feature embedding

Our model employs PLMs, including ProtBERT [19] and ESM-2 [20], for extracting contextual sequence representations. ProtBERT is a BERT-based PLM pre-trained on the UniRef100 dataset, producing an $L$  $\times $ 1,024 embedding matrix, where $L$ is the sequence length and each amino acid is represented by a 1,024-dimensional embedding vector. ESM-2 is a Transformer-based PLM using masked language modeling, yielding $L$  $\times $ 1,280 embeddings. These PLMs capture deep semantic and biophysical features, offering richer and more accurate feature representations compared to manual feature engineering.

#### Hierarchical feature extraction strategy

We design hierarchical feature extraction strategies for different prediction tasks. Both tasks utilize global protein features, while regulatory effect prediction requires additional local feature extraction, as phosphorylation often triggers conformational changes in specific regions that can allosterically regulate protein–protein binding affinities.

**Figure 3:**
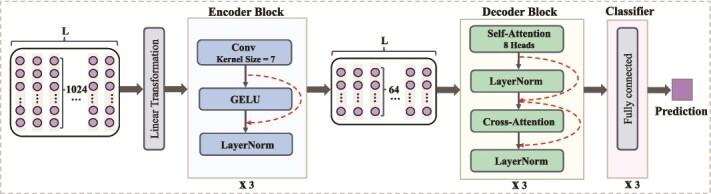
The architecture of DeepPhosPPI-1. It comprises three main modules: (a) Encoder, applying convolutional layers with GELU activation and LayerNorm to extract local features; (b) Decoder, employing multi-head self-attention and cross-attention to capture dependencies; (c) Classifier, using fully connected layers to project features for functional sites prediction. Red arrows denote residual connections. The embedding dimension is 1024 for ProtBERT (shown) and 1280 for ESM-2.

For functional phosphorylation sites identification, we adopt an end-to-end feature learning approach. The global representations are processed through a hierarchical convolutional structure comprising 3 convolutional layers to progressively expand the receptive field to adaptively extract local phosphorylation-related information, as illustrated in the encoder block of Fig. 3. For regulatory effect classification, we design the STY-Sliding Window to focus on local features around phosphorylation sites (S, T, and Y). It extracts 15 residues upstream and downstream of the target residue, forming a fixed-length sequence of 31, with “*” padding applied when the length is insufficient. This feature representation preserves important contextual information for subsequent prediction tasks.

### Model architecture

We propose the DeepPhosPPI-1 and DeepPhosPPI-2 models to address the two-stage prediction problem of functional site identification and its impact on PPIs, constructing a comprehensive framework for phosphorylation sites functional analysis.

#### DeepPhosPPI-1

DeepPhosPPI-1 incorporates CNN with attention mechanisms. As presented in Fig. 3, it extracts local features and models contextual dependencies through self-attention and cross-attention. The input is a protein representation matrix $ X \in \mathbb{R}^{L \times 1024} $, where $L$ represents the sequence length and 1024 is the embedding dimension.

**Encoder Module.** The encoder module extracts local features around the target residue and its surrounding context. First, the input features are projected from 1024 dimensions to 64 dimensions through a linear transformation:


(1) \begin{align*}& H^{(0)} = X W_{p} + b_{p}, \quad W_{p} \in \mathbb{R}^{1024 \times 64}, \quad b_{p} \in \mathbb{R}^{64},\end{align*}

where $ X $ is the input feature matrix, $ W_{p} $ is a weight matrix, and $ b_{p} $ is a bias vector. The projected features are then processed by three stacked 1D convolutional layers (kernel size 7) to capture local patterns. Each convolutional layer is followed by GELU activation function, which is defined as:


(2) \begin{align*}& \text{GELU}(x) = x \Phi(x), \quad \Phi(x) = \frac{1}{2} \left[ 1 + \text{erf} \left( \frac{x}{\sqrt{2}} \right) \right],\end{align*}

where $ \Phi (x) $ is the Gaussian error function, and the error function $\mathrm{erf}(x)$ is defined as:


(3) \begin{align*}& \mathrm{erf}(x) = \frac{2}{\sqrt{\pi}} \int_{0}^{x} e^{-t^{2}} \, dt,\end{align*}

where $t$ is the integration variable. To enhance model stability and preserve critical information, each convolutional layer incorporates LayerNorm and residual connections, which normalize feature distributions and alleviate gradient vanishing.

**Decoder Module.** The decoder module employs a multi-head attention mechanism to capture contextual dependencies in the input features. For each attention head, the attention weights are calculated as:


(4) \begin{align*}& \operatorname{Attention}(Q, K, V) = \operatorname{softmax} \left( \frac{Q K^{T}}{\sqrt{d_{k}}} \right) V,\end{align*}

where $ Q $, $ K $, and $ V $ are the query, key, and value matrices derived from the input features, and $ d_{k} $ is the dimension of the key vectors. The cross-attention layer further integrates the encoder outputs $ K $ and $ V $ with the target residue features $ Q $, enabling feature fusion between the encoder and the target site. The attention weights are computed as:


(5) \begin{align*}& \text{CrossAttention}(Q_{d}, K_{e}, V_{e}) = \text{softmax}\left(\frac{Q_{d} K_{e}^{T}}{\sqrt{d_{k}}}\right)V_{e}.\end{align*}

Specifically, in the cross-attention mechanism, the query matrix $Q_{d}$ is derived from the decoder’s intermediate representation, while the key and value matrices $K_{e}$ and $V_{e}$ are taken from the encoder output. Here, $d_{k}$ is the dimension of the key vectors.

**Classifier.** Finally, three feed-forward layers perform feature projection and classification, as:


(6) \begin{align*}& y = \operatorname{softmax} (W_{f} H + b_{f}), \quad W_{f} \in \mathbb{R}^{64 \times 2}, \quad b_{f} \in \mathbb{R}^{2},\end{align*}

where $ W_{f} $ and $ b_{f} $ are the weight matrix and bias vector, and $ y $ represents the probability distribution of whether the target residue is a functional phosphorylation site.

#### DeepPhosPPI-2

DeepPhosPPI-2 is an ensemble model comprising AttCNN-PhosPPI and Transformer-PhosPPI to predict the regulatory effect of phosphorylation on PPIs, as illustrated in Fig. 1. The input of the model includes the local features of protein $A$ (centered on the functional site $p$), the global features of protein $A$ and $B$. AttCNN-PhosPPI combines a CNN with an attention mechanism to extract local features, while Transformer-PhosPPI employs a Transformer-based architecture to capture long-range dependencies. These two submodels independently generate predictions and then integrate through voting (different voting methods are compared in the experimental result section).

#### AttCNN-PhosPPI

**Figure 4:**
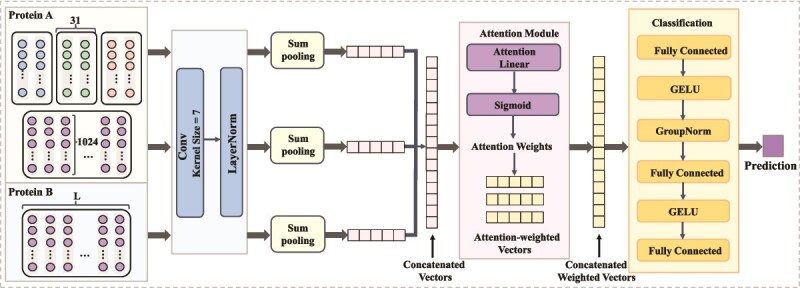
The AttCNN-PhosPPI architecture of the DeepPhosPPI-2. Local and global features of Protein $A$ and global features of Protein $B$ are processed through convolutional layers, followed by LayerNorm. Sum pooling aggregates the features, which are concatenated and passed to an attention module that computes attention scores through a linear layer and Sigmoid activation. Weighted features are then fed into a classifier comprising fully connected layers, GELU activations, and GroupNorm, outputs the final prediction.The embedding dimension is 1024 for ProtBERT (shown) and 1280 for ESM-2.

AttCNN-PhosPPI integrates CNN-based feature extraction and an attention mechanism to model phosphorylation-regulated protein interactions, as presented in Fig. 4.

#### Feature extraction

The input features are processed through a convolutional layer to extract local features, with the convolution operation formulated as:


(7) \begin{align*}& H_{j} = \phi\left(\text{Conv}(W_{j}, X)\right)\!,\end{align*}

where $ W_{\text{j}} $ is the convolution kernel weights and $ \phi (\cdot ) $ is a non-linear activation function. Subsequently, layerNorm is applied to normalize the feature maps, followed by sum pooling to integrate key information.

#### Attention module

The concatenated feature vector is fed into a linear attention layer and a Sigmoid activation function to compute a scalar weight:


(8) \begin{align*}& \alpha = \sigma\left(W_{\alpha} [h_{l}; h_{g}^{A}; h_{g}^{B}] + b_{\alpha}\right)\!,\end{align*}

where $h_{l}$, $h_{g}^{A}$, and $h_{g}^{B}$ denote the local and global features of proteins $A$ and $B$, respectively, $\sigma (\cdot )$ denotes the Sigmoid function, $W_{\alpha }$ and $b_{\alpha }$ are the learnable weight and bias. The scalar $\alpha \in (0, 1)$ adaptively controls the contribution of each component, resulting in the final weighted feature vector:


(9) \begin{align*}& h = \alpha h_{l} + \alpha h_{g}^{A} + (1 - \alpha) h_{g}^{B}.\end{align*}

This process assigns higher weights to informative features, enhancing key information.

#### Classifier

The weighted feature vector is fed into fully connected layers for classification, where GELU activation function and GroupNorm are applied to enhance non-linear representation and stability, ultimately outputting the regulatory effect.

#### Transformer-PhosPPI

**Figure 5:**
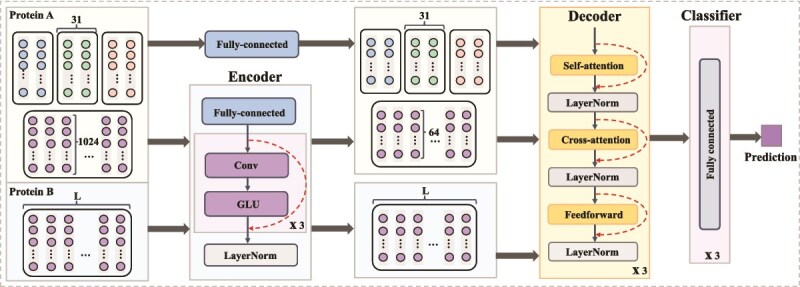
The Transformer-PhosPPI architecture of the DeepPhosPPI-2 model. It comprises three main components: (a) Encoder, where local and global features of Protein $A$ and global features of Protein $B$ are processed through a fully connected layer, and global features are further fed into 1D convolutional, GLU activation, and LayerNorm, with residual connections; (b) Decoder, employing multi-head self-attention and cross-attention to integrate information between Protein $A$ and Protein $B$, followed by a feed-forward network with residual connections and LayerNorm; (c) Classifier, composed of three fully connected layers to generate the classification. The embedding dimension is 1024 for ProtBERT (shown) and 1280 for ESM-2.

Transformer-PhosPPI adopts a Transformer architecture with multi-head self-attention and cross-attention to capture both local and global dependencies. It consists of an encoder, decoder, and classifier (Fig. 5).

#### Encoder

The encoder maps the local and global features of Protein $A$ and the global features of Protein $B$ into a 64-dimensional space through a fully connected layer. Next, these features are refined using convolutional blocks comprising a 1D convolution, gated linear unit (GLU) activation, and residual connections, which preserve critical information while suppressing noise. The GLU activation is given by:


(10) \begin{align*}& X_{\mathrm{GLU}} = (XW_{1} + b_{1}) \odot \sigma(XW_{2} + b_{2}),\end{align*}

where $ X $ is the input features, $ W_{1}, W_{2} $ and $ b_{1}, b_{2} $ are trainable parameters. The residual connections retain essential features. Finally, LayerNorm is applied to stabilize the feature distribution.

#### Decoder

The decoder processes local features and integrates them with the encoded global features. It consists of three consecutive computational blocks, each containing three key components, as follows. (i) The decoder employs multi-head self-attention to capture long-range dependencies within the local features, computed as in DeepPhosPPI-1 (see Equ. (4)). (ii) The decoder applies cross-attention to integrate local and global features, enhancing feature representation, computed as:


(11) \begin{align*}& \text{CrossAttention}(Q_{l}, K_{g}, V_{g}) = \text{softmax} \left( \frac{Q_{l} K_{g}^{T}}{\sqrt{d_{k}}} \right) V_{g},\end{align*}

where $ Q_{l} $ is derived from the local features, and $ K_{g} $ and $ V_{g} $ are obtained from the encoded global features. Each attention module is followed by residual connections and LayerNorm. (iii) The decoder incorporates a feed-forward network to apply nonlinear transformations to the features, followed by LayerNorm.

#### Classifier

The decoder outputs are processed by a three-layer fully connected network to predict whether the phosphorylation enhances or inhibits PPIs.

### Implementation

Protein sequences are the only input to our deep learning model and are encoded using PLM embeddings, with support for ProtBERT and ESM-2. For TASK1, we employ the cross-entropy loss during training. For TASK2, we implement a batch-weighted loss to address class imbalance, ensuring that the model pays adequate attention to minority class samples. RAdam optimizer combined with lookahead are utilized for training. The parameter configurations are: learning rate: 5e-4, batch size: 128, STY sliding window size: 31, multi-head self-attention: 8 heads, and kernel size of CNN: 7. Our network is implemented with PyTorch, trained on an NVIDIA A100 Tensor Core GPU.

## Experimental results

### Effectiveness of protein language model feature embedding

To evaluate the effectiveness of PLM feature embeddings, we compare ProtBERT and ESM-2 with traditional biological features in Task 1: Functional Phosphorylation Sites Identification. We select physical properties, PSSM, and protein one-hot encoding to construct a 47-dimensional traditional biological feature group (TraBIO). These features are widely used in protein function predictions [17, 23], as they encompass critical physicochemical, evolutionary, and sequence information within proteins, providing a comprehensive biological profile.

**Figure 6:**
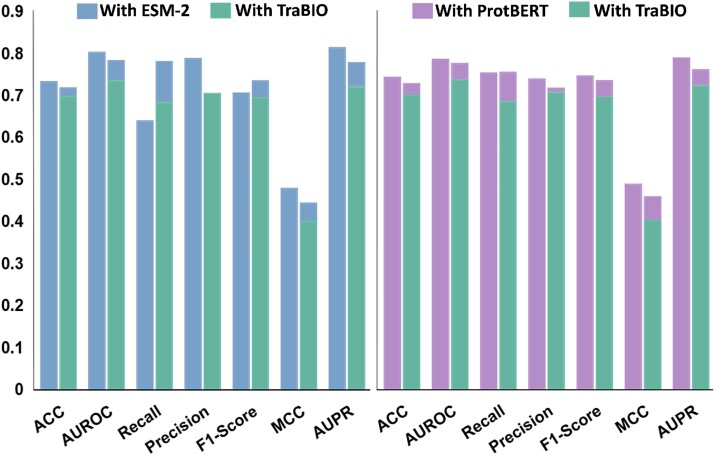
Prediction performances of the DeepPhosPPI-1 model using different types of features.

From Fig. 6 and Supplementary Table S2, it can be observed that PLMs, including ProtBERT and ESM-2 embeddings significantly outperform TraBIO in almost all evaluation metrics, highlighting the ability to capture essential biological information. Specifically, ProtBERT achieves the highest performance in Accuracy (0.745), F1-score (0.748), and MCC (0.491), while ESM-2 achieves the best AUROC (0.807), AUPR (0.818), and precision (0.792). These results demonstrate that PLMs exhibit reliable classification and strong robustness, highlighting superior capability in distinguishing functional phosphorylation sites from non-functional ones. Furthermore, incorporating PLM embeddings into TraBIO significantly enhances performance. After combining ProtBERT with TraBIO, AUPR and recall improve to 0.763 and 0.757 (an increase of 5.2% and 10.0% over TraBIO, respectively). AUROC and F1-score reach 0.778 and 0.737, which are 5.3% and 5.6% higher than TraBIO, though slightly lower than using ProtBERT alone. MCC improves to 0.461 (a 13.8% increase over TraBIO), indicating enhanced classification balance. A similar trend is observed for ESM-2: while recall improves to 0.785 after integrating with TraBIO, other metrics such as MCC and precision exhibit a slight decline. Surprisingly, the combined approaches do not consistently outperform the standalone ProtBERT and ESM-2 embeddings, suggesting that PLM features sufficiently capture key biological information, and incorporating TraBIO may introduce redundancy.

To clearly compare the discriminative capacities of different feature types, we perform t-SNE visualization. As illustrated in Supplementary Fig. S1, ProtBERT and ESM-2 features exhibits distinct clusters with clear boundaries between functional and non-functional sites, while TraBIO features show substantial overlap and disorganized distribution. Incorporating PLMs features enhances inter-class separability with localized clustering and boundary emergence, although overlaps still exist in transitional areas. The visualization results consistent with the quantitative evaluation. Overall, experimental results confirm that PLM embeddings effectively capture residue preferences, sequence conservation, and implicit structural information, providing a comprehensive biological feature representation.

### Performance evaluation of the functional phosphorylation sites identification

**Figure 7:**
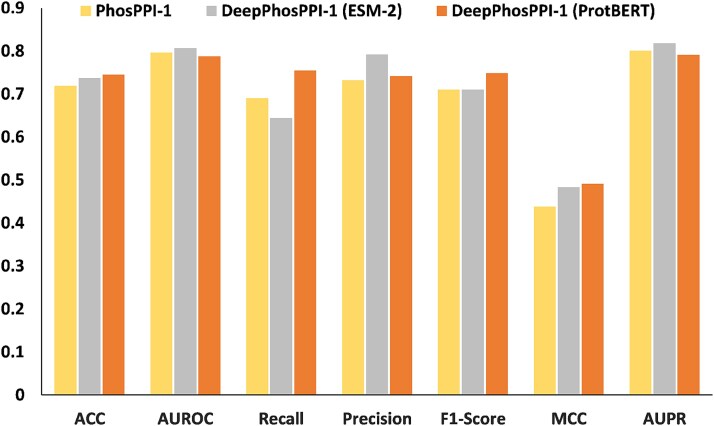
Performance evaluation of the Functional Phosphorylation Sites Identification.

To evaluate model performance, we compare DeepPhosPPI-1 with competing method PhosPPI-1 [12], which is currently the most competitive approach for functional phosphorylation sites identification. DeepPhosPPI-1 employs an attention-based CNN DL architecture with PLM embeddings (ProtBERT or ESM-2), while PhosPPI-1 applies LightGBM for prediction. The test set is derived from DatasetA (see Materials and Methods). Figure 7 and Supplementary Table S3 present the evaluation results.

It is clear that while the AUROC (0.788) and AUPR (0.791) scores of DeepPhosPPI-1 (ProtBERT) are slightly lower than those of PhosPPI-1 (0.796 and 0.801, respectively), DeepPhosPPI-1 (ESM-2) achieves higher scores of 0.807 and 0.818 on AUROC and AUPR, indicating better model ranking performance. Apart from AUROC and AUPR, DeepPhosPPI-1 consistently outperforms PhosPPI-1 across all other evaluation metrics. Specifically, DeepPhosPPI-1 (ProtBERT) achieves 0.745 in Accuracy, which is 3.6% higher than PhosPPI-1 (0.719), demonstrating better overall predictive performance. Its Recall reaches 0.755, which is 9.4% higher than PhosPPI-1 (0.690), highlighting its stronger ability to identify functional phosphorylation sites while reducing false negatives. High Accuracy and Recall provide reliable candidate sites for downstream regulatory effect prediction. DeepPhosPPI-1 (ProtBERT) also attains an F1-score of 0.748 and MCC of 0.491, which are 5.4% and 12.1% higher than PhosPPI-1 (0.710 and 0.438), further indicating better classification balance. While the DeepPhosPPI-1 (ESM-2) shows slightly lower F1-score (0.710) and MCC (0.483) than DeepPhosPPI-1 (ProtBERT), it achieves the highest Precision (0.792) among all methods, demonstrating superior specificity and reducing unnecessary biological validation and improving research efficiency. ROC and PR curves are provided for an intuitive comparison. as shown in Supplementary Fig. S2, DeepPhosPPI-1 (ESM-2) demonstrates overall superior performance. Notably, DeepPhosPPI-1 (ProtBERT) exhibits comparable advantages to DeepPhosPPI-1 (ESM-2) in the false positive rate of 0.2–0.4 on the ROC curve and in the recall range of 0.5–0.8 on the PR curve. From these results, it is evident that DeepPhosPPI-1 demonstrates superior performance in functional phosphorylation sites identification. Additionally, we evaluate the performances of other PLMs, including ESM2 with varying parameter scales [24] and SaProt [25], with detailed results presented in Supplementary Table S4. DeepPhosPPI-1 framework supports flexible substitution of feature embedding modules, facilitating adaptation to various downstream applications and emerging techniques.

### Performance evaluation of regulatory effect classification on PPIs

To assess the performance of our method in Regulatory Effect Classification for PPIs, we compare our DeepPhosPPI-2 with PhosPPI-2 [12], Betts [11], and FoldX [10]. PhosPPI-2 is a sequence-based machine learning method that employs the LightGBM model for prediction. Betts predicts based on residue pair potential energy changes, while FoldX relies on structural energy calculations. All models are evaluated on the Betts benchmark dataset [11] for a fair comparison. To further assess DeepPhosPPI-2’s performance, we also evaluate it on the independent test set, Dset_308. The results are summarized in Table 1.

**Table 1:** Performance comparison among DeepPhosPPI-2 and competing methods on the test sets

	Accuracy	AUROC	Recall	Precision	F1-Score	MCC	AUPR
Betts Test Set							
PhosPPI-2 [12]	0.726	0.627	**0.963**	0.733	0.833	0.196	0.806
Betts [11]	0.740	0.623	0.350	*N/A*	0.509	**0.526**	*N/A*
FoldX [10]	*N/A*	0.466	0.009	*N/A*	0.016	0.412	*N/A*
DeepPhosPPI-2 (ESM-2)	0.683	0.536	0.829	0.750	0.787	0.172	0.730
DeepPhosPPI-2 (ProtBERT)	**0.775**	**0.820**	0.903	**0.803**	**0.850**	0.411	**0.921**
Dset_308 Test Set							
PhosPPI-2 [12]	0.763	0.761	**0.958**	0.761	**0.848**	0.390	0.875
DeepPhosPPI-2 (ESM-2)	0.731	0.737	0.798	0.810	0.804	0.374	0.831
DeepPhosPPI-2 (ProtBERT)	**0.773**	**0.773**	0.817	**0.849**	0.833	**0.480**	**0.875**

Note: The predictions by PhosPPI-2 are generated from source codes. The results of Betts and FoldX are obtained from [12].

On the Betts benchmark dataset, DeepPhosPPI-2 (ProtBERT) achieves the best performance in Accuracy, AUROC, Precision, F1-score, and AUPR. Specifically, DeepPhosPPI-2 (ProtBERT) attains 0.775 in Accuracy, outperforming Betts (0.740) and PhosPPI-2 (0.726) by 4.7% and 6.7%, respectively. Its AUROC value reaches 0.820, exceeding PhosPPI-2 (0.627) by 0.193, Betts (0.623) by 0.197, and FoldX (0.466) by 0.354, representing an improvement of at least 30.8% and up to 76.0% over the competing methods. With a Precision of 0.803, DeepPhosPPI-2 (ProtBERT) surpasses PhosPPI-2 by 9.6%, effectively reducing false positives and enhancing classification reliability. We focus more on F1-score and AUPR, which better capture the model’s performance while balancing Precision and Recall. Its F1-score reaches 0.850, outperforming the competing methods by 2% (PhosPPI-2) to 97.5% (FoldX). DeepPhosPPI-2 (ProtBERT) achieves 0.921 in AUPR, which is 14.3% higher than that of PhosPPI-2, the second best method. DeepPhosPPI-2 (ProtBERT) ranks second in Recall and MCC, achieving 0.903 and 0.411, respectively, a little behind PhosPPI-2 (0.963) and Betts (0.526). Based on these findings, it is evident that DeepPhosPPI-2 (ProtBERT) yields the best overall performance, outperforming all the competing methods in Regulatory Effect Classification for PPIs. On the Dset_308 test set, DeepPhosPPI-2 (ProtBERT) achieves strong performance with an Accuracy of 0.773, AUROC of 0.773, Precision of 0.849, and MCC of 0.480. Consistently, our method achieves outperforming performance on both the Betts dataset and the Dset_308 test set, further validating DeepPhosPPI-2 (ProtBERT)’s strong robustness. We further provide ROC and PR curves on the Dset_308 and Betts test sets for an intuitive performance comparison. As shown in Supplementary Fig. S3 and Fig. S4, DeepPhosPPI-2 (ProtBERT) consistently outperforming other methods on the ROC and PR curves, showing strong overall discriminative ability

In addition, DeepPhosPPI-2 (ESM-2) exhibits lower overall performance than DeepPhosPPI-2 (ProtBERT) across both test sets, though it still achieves stable F1-scores of 0.787 and 0.804, and AUPRs of 0.730 and 0.831. This disparity may stem from differences in representation styles between the two PLMs and their compatibility with the ensemble DL architecture. The context-aware representations of ProtBERT may better capture sequence patterns relevant to regulatory effects, leading to superior classification performance. Therefore, in the subsequent study of predicting phosphorylation effects on PPIs, we adopt DeepPhosPPI-2 based on ProtBERT embeddings as the default model. Nonetheless, our framework remains flexible in supporting the substitution of embedding models, offering broad extensibility for future research and task transfer.

### The effects of global dependencies and local features

Previous studies have shown that CNNs can effectively extract local features [13] while Transformers excel at capturing long-range global dependencies [18], both crucial for regulating phosphorylation effects on PPIs. To explore the role of different features in DeepPhosPPI-2, we conduct an ablation study comparing Transformer-PhosPPI, AttCNN-PhosPPI, and the ensemble model. Results are shown in Table 2.

**Table 2:** Prediction performances of our DeepPhosPPI-2, Transformer-PhosPPI, and AttCNN-PhosPPI models

	Accuracy	AUROC	Recall	Precision	F1-Score	MCC	AUPR
Transformer-PhosPPI	**0.775**	0.748	0.899	0.806	**0.850**	0.414	0.858
AttCNN-PhosPPI	0.752	0.744	0.797	**0.844**	0.820	**0.423**	0.865
DeepPhosPPI-2	**0.775**	**0.820**	**0.903**	0.803	**0.850**	0.411	**0.921**

Transformer-PhosPPI effectively learns global dependencies, strengthening DeepPhosPPI-2’s overall effectiveness. It achieves 0.775, 0.899, and 0.850 in Accuracy, Recall, and F1-score respectively, demonstrating overall classification capability in this imbalanced task. However, its MCC (0.414) and AUPR (0.858) are slightly lower than those of AttCNN-PhosPPI, suggesting limited exploitation of local features. AttCNN-PhosPPI leverages CNN to extract local features, contributing to stable classification performance. It achieves a Precision of 0.844, MCC of 0.423, and AUPR of 0.865, all slightly higher than Transformer-PhosPPI, demonstrating better identification of enhancement phosphorylation sites while reducing the misclassification of inhibition ones. However, its Recall (0.797) is lower than Transformer-PhosPPI (0.899), indicating that the dependence on local features alone may limit detection of phosphorylation site regulatory functions.

DeepPhosPPI-2 ensembles Transformer-PhosPPI and AttCNN-PhosPPI to leverage global and local features, thus improving performance in regulatory effect classification for PPIs. It achieves the overall best performance, with AUROC (0.820) and Recall (0.903) surpassing Transformer-PhosPPI and AttCNN-PhosPPI and demonstrating its enhanced classification sensitivity. Its AUPR (0.921) also significantly exceeds the submodels (0.858 and 0.865), confirming its robustness. These findings indicate that integrating global and local features enables DeepPhosPPI-2 achieves superior predictive performance, demonstrating ensemble learning as an effective strategy for regulatory effect classification for PPIs.

### The effect of voting mechanisms on ensemble prediction performance

To identify the most effective ensemble strategy for improving regulatory effect classification for PPIs, we compare three voting mechanisms, including Soft Voting, Hard Voting, and Max Confidence Voting, and evaluate their impact on the performance of DeepPhosPPI-2.

**Figure 8:**
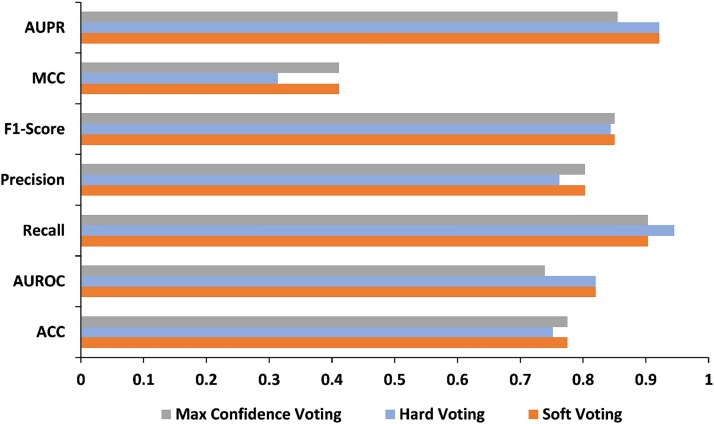
Performance evaluation of three different voting mechanisms.

From Fig. 8 and Supplementary Material Table S5, it is clear that Soft Voting ranks first in six out of the seven evaluation metrics, achieving 0.775, 0.850, 0.820, 0.803, 0.411, and 0.921 in Accuracy, F1-score, AUROC, Precision, MCC, and AUPR, respectively. This suggests that the Soft Voting strategy effectively integrates the complementary strengths of AttCNN-PhosPPI and Transformer-PhosPPI, enabling robust predictions under class imbalance. Hard Voting achieves 0.945 in Recall, which is higher than Soft Voting’s 0.856. In contrast, its Precision, Accuracy, and MCC are lower by 0.041, 0.028, and 0.097, respectively. Max Confidence Voting achieves similar Accuracy and F1-score as Soft Voting, although its AUROC and AUPR are 0.739 and 0.855, which are lower than Soft Voting by 0.081 and 0.066, indicating limited discriminative capability, which is potentially caused by overconfident decisions near classification boundaries. Overall, the superior and reliable performance of Soft Voting substantiates its designation as the default fusion strategy in DeepPhosPPI-2, while the relatively small performance differences across the voting mechanisms suggest that the model is robust to integration strategies.

### Case study

To visualize DeepPhosPPI predictions, we select the BAD protein (Uniprot ID: Q92934) as a case study to show predicted phosphorylation sites involved in PPI regulation. BAD regulates cell survival and apoptosis by interacting with partner proteins [26, 27]. Abnormal BAD phosphorylation disrupts this balance, contributing to diseases such as cancer and neurodegenerative disorders [28, 29]. Therefore, accurately predicting the regulatory effects of BAD phosphorylation on PPIs is essential for targeted therapeutic development.

#### Prediction of BAD phosphorylation sites involved in PPI regulation

**Figure 9:**
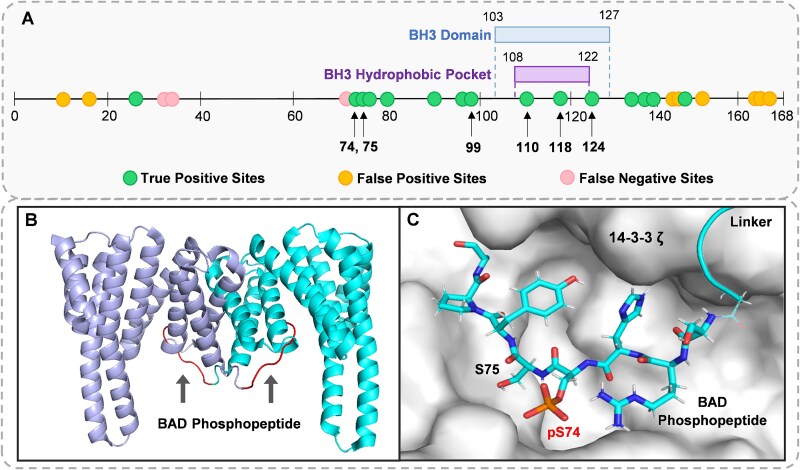
BAD phosphorylation sites analysis and BAD–14-3-3$\zeta $ structural interaction. (A) Distribution of potential phosphorylation sites and functional regions in BAD. BH3 domain and hydrophobic pocket are marked with blue and purple boxes, respectively. Green, yellow, and pink dots indicate true positives, false positives, and false negatives. Key phosphorylation sites are shown in bold with arrows. (B) 3D structure of BAD-14-3-3$\zeta $ complex (7Q16), with the 14-3-3$\zeta $ dimer shown in cyan and purple. The BAD phosphopeptide is bound within its hydrophobic groove (indicated by arrows). (C) Binding interface between the BAD phosphopeptide and 14-3-3$\zeta $, where phosphorylated Ser74 is highlighted in red and positioned within the binding pocket of 14-3-3$\zeta $.

In this case study, DeepPhosPPI identifies 15 potential phosphorylation sites regulating PPIs, as shown in Fig. 9A. Experimental results in Supplementary Table S6 show that DeepPhosPPI achieves 0.706, 0.774, 0.682, 0.750, 0.413, and 0.803 in Accuracy, AUROC, Precision, F1-score, MCC, and AUPR, respectively, outperforming existing methods. These results further validate DeepPhosPPI’s advantages in predicting phosphorylation regulatory effects on PPIs.

Studies have demonstrated that phosphorylation at Ser74, Ser75, Ser99, and Ser118 regulates BAD PPIs [30–32]. Ser74/75 phosphorylation promotes BAD–14-3-3$\zeta $ binding and prevents its interaction with Bcl-2/Bcl-XL, suppressing apoptosis. Ser99 enhances 14-3-3 affinity, stabilizing inactive BAD. Ser118 regulates BAD–Bcl-2/Bcl-XL interaction and may influence BAD-14-3-3$\zeta $ binding in a similar way. The BH3 domain (103–127), forming an $\alpha $-helix, serves as a key apoptotic signaling region in the BCL-2 family [33], with its hydrophobic pocket (108–122) providing a critical PPI interface [34]. Our study identifies Tyr110, Ser118, and Ser124 within BH3 domain, and peripheral Ser99. Their phosphorylation may alter BH3 conformation, modulating BAD and 14-3-3$\zeta $ interactions. To further explore the understanding of dependencies between functional residues, we visualize attention weights among key phosphorylation sites. It can be observed from Supplementary Fig. S5 that Ser74, Ser75, Tyr76, and Ser99 constitute a spatially proximate regulatory segment with attention values in the 0.3-0.5 range, showing mutual dependency and suggesting a potential multi-site cooperative regulatory mechanism.

#### 7Q16 crystal structure analysis: BAD phosphorylation in 14-3-3$\zeta $ binding

To investigate how BAD phosphorylation modulates PPI, we examine the interaction between BAD and 14-3-3$\zeta $ using 7Q16 (PDB ID) crystal structure [32]. It reveals that phosphorylated Ser74 binds to the hydrophobic groove of 14-3-3 (Fig. 9B). 14-3-3$\zeta $ exists as a dimer, with its two subunits represented in cyan and purple, while the BAD phosphopeptide, shown in red, occupies both subunits’ hydrophobic pockets, forming a stable interaction interface.

Interface analysis (Fig. 9C) shows that phosphorylated Ser74 inserts into the 14-3-3$\zeta $ pocket, forming a stable complex through hydrogen bonds and electrostatic interactions. This phosphorylation may induce conformational rearrangements at the BAD binding interface, affecting its interactions with 14-3-3$\zeta $ and other PPI partners. Notably, Ser75 near the binding interface may functional complement Ser74 under specific conditions, collectively stabilizing the BAD–14-3-3$\zeta $ interaction. Our DeepPhosPPI model identifies potential BAD phosphorylation sites regulating PPIs, and visualizes their interaction with 14-3-3$\zeta $ using the 7Q16 crystal structure. These findings enhance understanding of BAD–14-3-3$\zeta $ regulation and provide insights for targeted interventions.

## Conclusions

Accurate prediction of the effects of phosphorylation on PPIs facilitates understanding protein biological functions. In this study, we propose DeepPhosPPI, a novel deep learning framework that integrates local and global features from protein sequences to predict phosphorylation-mediated regulatory effects on PPIs. It combines PLM embedding with an innovative ensemble architecture that fuses attention-based CNN and Transformer models, effectively capturing local sequence features and global dependencies critical for accurate prediction. Experimental results demonstrate that DeepPhosPPI outperforms existing methods in both identifying functional phosphorylation sites and classifying their regulatory effects. Our study shows that the proposed feature extraction and ensemble learning strategies effectively predict phosphorylation effects on PPIs. This framework is promising for understanding disease mechanisms and supporting therapeutic development, with potential applications in broader biological sequence analysis and prediction problems.

**Key Points**

- We propose DeepPhosPPI, the first DL framework with attention-CNN and Transformer for identifying phosphorylation effects on PPIs.
- We employ a hierarchical feature extraction strategy using multi-layer CNNs with expanded receptive fields and STY-sliding window.
- Experimental results show that DeepPhosPPI consistently outperforms SOTA methods in identifying functional sites and classifying regulatory effects.
- We construct and publicly release a dataset of phosphorylation effects on PPIs.

## Supplementary Material

supplymentary-materials_Revision2_bbaf462

## Data Availability

The source codes are available at https://github.com/YyinGong/DeepPhosPPI/tree/main. The datasets used in this study can be downloaded from https://github.com/YyinGong/DeepPhosPPI/tree/main/Datasets
