# Supplementary material for: DeepPhosPPI: a deep learning framework with attention-CNN and transformer for predicting phosphorylation effects on protein–protein interactions
Source: Brief Bioinform. 2025 Sep 7;26(5):bbaf462. doi: 10.1093/bib/bbaf462 (PMC12414479; doi:10.1093/bib/bbaf462)
Supplement: supplymentary-materials_Revision2_bbaf462 [file supplymentary-materials_revision2_bbaf462.pdf]

Table S1. Statistics of the datasets used in this work.

Dataset	Non-functional sites	Functional sites	Enhancement sites	Inhibition sites
DatasetA Train set	1722	1722	N/A	N/A
DatasetA Test set	432	432	N/A	N/A
DatasetB Train set	0	2775	1930	845
Betts Test set	0	306	217	89
Dset_308 Test set	0	308	213	95

Table S2. Prediction performances of DeepPHOPPI-1 model using different features.

Features	ACC	AUROC	Recall	Precision	F1-Score	MCC	AUPR
ProtBERT	**0.745**	0.788	0.755	0.741	**0.748**	**0.491**	0.791
ESM-2	0.737	**0.807**	0.644	**0.792**	0.710	0.483	**0.818**
TraBIO	0.703	0.739	0.688	0.709	0.698	0.405	0.725
ProtBERT+TraBIO	0.730	0.778	0.757	0.719	0.737	0.461	0.763
ESM-2+TraBIO	0.722	0.787	**0.785**	0.698	0.739	0.448	0.782

Note: ProtBERT and ESM-2 represent the feature embedding obtained from ProtBERT and ESM-2 respectively. TraBIO refers to traditional biological feature group (TraBIO), which includes Physical Properties, Position-Specific Scoring Matrix (PSSM), and protein one-hot embedding.

Table S3. Performance comparison of the functional Phosphorylation Sites Identification with SOTA Method.

Methods	ACC	AUROC	Recall	Precision	F1-Score	MCC	AUPR
PhosPPI-1	0.719	0.796	0.690	0.732	0.710	0.438	**0.801**
DeepPHOPPI-1 (ESM-2)	0.737	**0.807**	0.644	**0.792**	0.710	0.483	**0.818**
DeepPHOPPI-1 (ProtBERT)	**0.745**	0.788	**0.755**	0.741	**0.748**	**0.491**	0.791

Note: The predictions by PhosPPI-1 are generated from source codes.

Table S4. Performance comparison using different feature embedding methods on the test set.

Methods	ACC	AUROC	Recall	Precision	F1-Score	MCC	AUPR
Saprot	0.590	0.624	**0.810**	0.563	0.664	0.201	0.620
DeepPHOPPI-1 (ESM-2_{150M})	0.722	0.787	0.697	0.697	0.738	0.448	0.781
DeepPHOPPI-1 (ESM-2_{650M})	0.737	**0.807**	0.644	**0.792**	0.710	0.483	**0.818**
DeepPHOPPI-1 (ProtBERT)	**0.745**	0.788	0.755	0.741	**0.748**	**0.491**	0.791

Note: The predictions using ESM-2 in the main text refer to ESM-2_{650M} by default.

Table S5. Prediction performances of DeepPHOPPI-2 model using different Ensemble Voting Methods.

Features	ACC	AUROC	Recall	Precision	F1-Score	MCC	AUPR
Soft Voting	**0.775**	**0.820**	0.903	**0.803**	**0.850**	**0.411**	**0.921**
Hard Voting	0.752	0.820	**0.945**	0.762	0.844	0.314	**0.921**
Max Confidence Voting	**0.775**	0.739	0.903	**0.803**	**0.850**	**0.411**	0.855

Table S6. Prediction performance of Protein BAD (Uniprot ID: Q92934).

Methods	ACC	AUROC	Recall	Precision	F1-Score	MCC	AUPR
PhosPPI-1	0.677	0.681	**0.889**	0.640	0.742	0.369	0.748
DeepPHOPPI-1	**0.706**	**0.774**	0.833	**0.682**	**0.750**	**0.413**	**0.803**

Note: The predictions by PhosPPI-1 are generated from source codes.

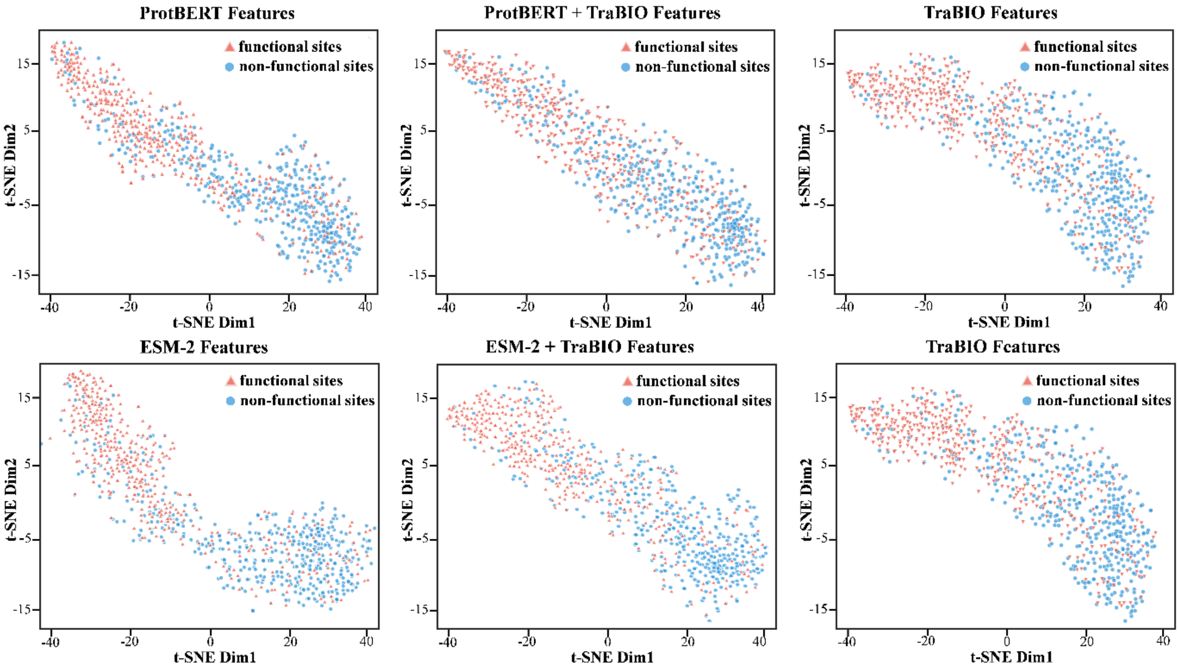

Fig. S1. t-SNE visualization using different types of features.

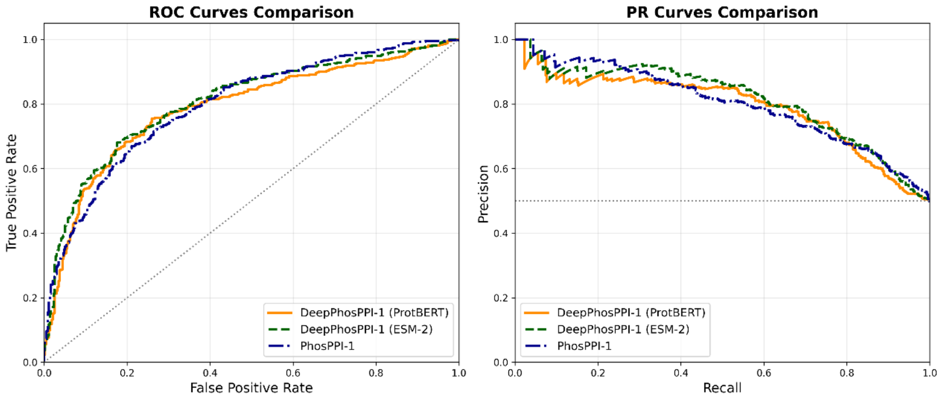

Fig. S2. Comparison of ROC and PR Curves for Functional Phosphorylation Site

Identification.

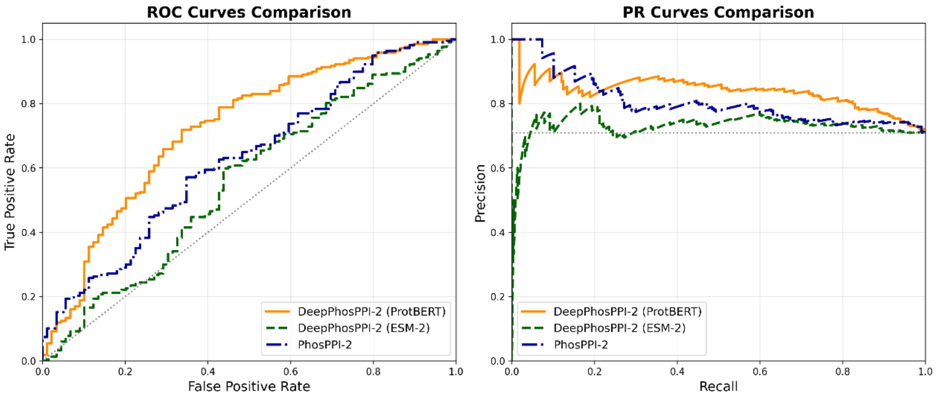

Fig. S3. Comparison of ROC and PR Curves on the Betts Test Set for Regulatory Effect Classification.

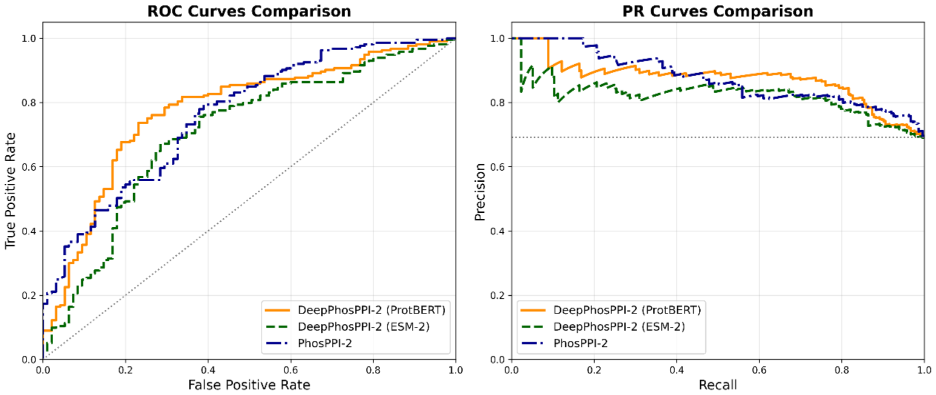

Fig. S4. Comparison of ROC and PR Curves on the Dset_308 Test Set for Regulatory Effect Classification.

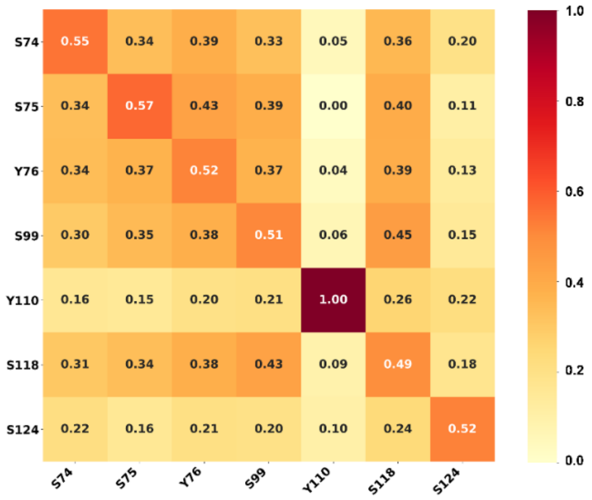

Fig. S5. Attention Weights Between Key BAD Phosphorylation Sites
